# Intravitreal Dexamethasone Implant at the Time of Silicon Oil Removal to Treat Persistent Macular Edema after Rhegmatogenous Retinal Detachment Repair

**DOI:** 10.3390/jcm12041697

**Published:** 2023-02-20

**Authors:** Francesco Pignatelli, Annalisa Nacucchi, Alfredo Niro, Samuele Gigliola, Fedele Passidomo, Rossella Donghia, Giuseppe Addabbo

**Affiliations:** 1 Eye Clinic, Hospital “SS. Annunziata”, ASL Taranto, 74100 Taranto, Italy; 2 Unit of Research Methodology and Data Sciences for Population Health, “Salus in Apulia Study” National Institute of Gastroenterology “Saverio de Bellis” Research Hospital, Castellana Grotte, 70013 Bari, Italy

**Keywords:** macular edema, rhegmatogenous retinal detachment, vitrectomy, intravitreal dexamethasone implant

## Abstract

Background: An intravitreal dexamethasone implant (DEX-I) was found to be effective and safe for the treatment of cystoid macular edema (CME) after vitrectomy for rhegmatogenous retinal detachment (RRD) and in silicone oil (SO)-filled eyes. We aimed to investigate the efficacy and safety of DEX-I at the time of SO removal for the treatment of recalcitrant CME after successful RRD repair. Methods: A retrospective review of the medical records was performed on 24 consecutive patients (24 eyes) with recalcitrant CME after RRD repair who were treated with a single 0.7-mg DEX-I at the time of SO removal. The main outcome measures were changes in best-corrected visual acuity (BCVA) and central macular thickness (CMT). A regression model was performed to assess the relationship between BCVA and CMT at 6 months and independent variables. Results: In all 24 patients, CME occurred after RRD repair and remained despite topical therapy. The mean time of CME onset was 27.4 ± 7.7 days after vitrectomy. The mean time between vitrectomy and DEX-I was 106.8 ± 10.1 days. The mean CMT was significantly decreased from 429.6 ± 59.1 µm at baseline to 294 ± 46.4 µm at month 6 (*p* < 0.0001). The mean BCVA significantly improved from 0.99 ± 0.3 at baseline to 0.60 ± 0.3 at month 6 (*p* < 0.0001). An elevation of intraocular pressure was observed in one (4.1%) eye, which was medically managed. A univariate regression model revealed a relationship between month-6 BCVA after DEX-I and gender (β = −0.27; *p* = 0.03) and macular status (β = −0.45; *p* = 0.001) when RRD occurred. No relationship between month-6 CMT and independent variables was found. Conclusions: DEX-I at the time of SO removal had an acceptable safety profile and achieved favorable outcomes in eyes affected by recalcitrant CME that occurred after RRD repair. RRD-related macular status is significantly associated with visual acuity after DEX-I.

## 1. Introduction

Cystoid macular edema (CME) is a primary cause of visual impairment following different ophthalmic surgical procedures, including successful vitreoretinal surgery [1,2]. Its onset may occur weeks after vitrectomy [1,2], even after a successful vitrectomy for rhegmatogenous retinal detachment (RRD) [3,4,5], and it may evolve into a chronic condition [1,2,3,4,5]. CME occurred in 9.6% to 25.2% of the eyes following RRD repair [3,4,5]. In the multifactorial pathophysiology of CME [6], subclinical inflammation is the real cause of CME after vitrectomy for RRD [7].

Furthermore, the silicone oil (SO) endotamponade, used in the most challenging cases of RRD or in every case that requires a long-term filling effect [8], may cause the occurrence of CME [9,10], with an incidence that was estimated between 19.6% and 45% of SO-filled eyes [9,10,11]. Furthermore, the duration of SO tamponade can correlate with CME onset [9,12,13]. The main pathogenetic mechanisms of SO-related complications involve direct acute cytotoxic effects, chemotaxis of inflammatory cells against SO emulsification, and the accumulation of proinflammatory cytokines in the fluid between SO and the retina [9,12,13,14,15].

Topic nonsteroidal anti-inflammatory drug (NSAID) category is typically used as the earliest and most common approach [16,17], but an early combination of NSAIDs and steroids appeared to offer benefits over monotherapy for acute CME [18,19]. Periocular or intravitreal corticosteroid injections and intravitreal Vascular Endothelial Growth Factor inhibitors (anti-VEGF) have emerged as effective options for managing CME when it becomes recalcitrant [2]. Recently, an intravitreal dexamethasone implant (DEX-I) was found to be effective and safe for the treatment of CME after vitrectomy for RRD [20,21,22,23,24] and in SO-filled eyes [25]. However, in a recent case report, DEX-I showed less effectiveness in the treatment of macular edema in a SO-filled eye than that in an eye after SO removal [26].

In this study, we investigated the efficacy and safety of DEX-I at the time of SO removal in vitrectomized eyes for RRD who developed unresponsive to medical therapy CME.

## 2. Materials and Methods

### 2.1. Design

This retrospective study included eyes with CME occurring after repair of RRD by vitrectomy with SO (Siluron 1000 centistokes, Fluoron, Ulm, Germany) tamponade and refractory to topical therapy who underwent 0.7-mg DEX-I (OzurdexTM; Allergan, an Abbvie company, Irvine, CA, USA) at the time of SO removal, between March 2019 and March 2020 at the Eye Clinic, Hospital “SS. Annunziata”, Taranto, Italy.

The Internal Review Board (IRB) of the Eye Clinic of Hospital “SS. Annunziata” approved the study on March 2019. Informed consent was obtained from all individual participants included in the study before surgery and before DEX-I. The tenets of the Declaration of Helsinki were regarded.

### 2.2. Patients

Patient selection was based on visual impairment due to CME occurring after vitrectomy. Patients had previously undergone an extensive vitrectomy and SO injection for RRD and were discharged with topical therapy, including NSAIDs and steroid eye drops.

CME was defined as a central macular thickness (CMT) > 250 µm with cystoid spaces between retinal layers. Refractory CME was defined as a lack of therapeutic response for at least three months. CME that did not adequately respond to NSAIDs and steroids was defined as a CMT > 250 µm or less than 10% CMT reduction and a visual acuity improvement ≤ 0.1 LogMAR. A period of at least 3 months was required from vitrectomy to SO removal. In all the cases, DEX-I was administered at the time of SO removal. Ocular hypertension was defined as intraocular pressure (IOP) ≥ 25 mmHg without any ocular hypotensive medication.

### 2.3. Inclusion/Exclusion Criteria

Patients with a SO-filled eye and CME who did not adequately respond to previous treatment with topical diclofenac sodium and betamethasone drops 4 times a day for at least 3 months and agreed to receive a DEX-I were included in the study. CME was diagnosed by Optical Coherence Tomography (OCT, Cirrus HD-OCT 5000, Carl Zeiss Meditec Inc., Oberkochen, Germany). Patients were excluded if they had any other retinal pathology, a history of ocular hypertension, glaucoma, uveitis, or any other intraocular surgery after the vitrectomy.

### 2.4. Assessments

Each patient underwent a complete ophthalmic evaluation, including best-corrected visual acuity (BCVA) assessment by the Early Treatment Diabetic Retinopathy Study (ETDRS) protocol, CMT measurement by OCT, fundoscopy, and IOP measurement with a Goldmann applanation tonometer. For statistical purposes, ETDRS values were converted to the logarithm of the minimum angle of resolution (logMAR). CMT was defined as the average thickness of the macula in the central 1 mm ETDRS grid. Follow-up visits were performed on days 30, 60, and 90 (±5 days). BCVA, CMT, and IOP were collected at all the follow-up visits. 

### 2.5. SO Removal and DEX-I

Below, we have reported the procedures performed in all cases. A topical iodopovidone solution (0.6%) was used 3 times daily for 3 days before SO removal and DEX-I. 25G 3 ports vitrectomy was performed using the Constellation^®^ Vision System (Alcon Laboratories, Fort Worth, TX, USA). Povidone-iodine 5% preparation (Oftasteril, Alfa Intes Industria Terapeutica Splendore S.r.l., Naples, Italy) was applied to the cornea, conjunctival sac, and periocular skin for 3 min before surgery. Peribulbar anesthesia was performed on all patients. Conjunctival displacement with forceps and three 30% oblique incisions 3.5 cm from the limbus were performed to insert three valve cannula trocar systems. Oil aspiration was performed with a 25G cannula and Alcon aspiration device. Fluid-air exchanges were then performed until no more oil droplets were microscopically observable. Furthermore, the eye was filled with a balanced saline solution (BSS). For posterior visualization, Oculus BIOM 4 (Oculus Surgical Inc., Port St. Lucie, FL, USA) was used. Finally, DEX-I was implanted via pars plana directly in the inferotemporal quadrant using the 22-gauge applicator device. All patients were placed on topical antibiotic/steroid therapy for four times daily for seven days after surgery. All surgeries were performed by a single surgeon (FP).

### 2.6. Statistical Analysis

The qualitative variables are presented as frequencies and percentages, while the quantitative data is presented as means ± standard deviations. No formal sample size calculation was performed. For assessing the change in BCVA and CMT over follow-up, the non-parametric test known as the Wilcoxon rank-sum test was used. All statistical tests were performed at the *p* < 0.05 significance level. Univariate and multivariate regression models were performed to assess the relationship between BCVA and CMT at 6 months after DEX-I and each independent variable. The independent variables included gender, age, lens status, glaucoma disease, RRD-macular status, days between vitrectomy and CME onset, time duration of topical therapy, including NSAIDs and steroids, and days between vitrectomy and DEX-I. Statistical analysis was made using STATA 12.1 Statistical Software (StataCorp), 2014, release 12 (College Station, TX, USA).

## 3. Results

Twenty-four eyes of 24 consecutive patients who underwent vitrectomy for RRD and SO endotamponade and developed a CME that did not adequately respond to topical diclofenac and betamethasone for a mean treatment period of at least 3 months after vitrectomy for RRD repair were included in the study. The age of patients ranged from 55 years to 88 years. Eight (33.3%) patients were women. All phakic patients had undergone cataract surgery at the same time as a vitrectomy. The time from vitrectomy to CME onset ranged from 14 to 42 days, and the time for DEX-I after vitrectomy ranged from 91 to 115 days. Demographic and clinical characteristics are summarized in Table 1. 

After DEX-I, BCVA significantly improved from 0.99 ± 0.28 Log MAR at baseline to 0.62 ± 0.3 LogMAR at 1 month and remained stable over six months (*p* < 0.0001). CMT significantly decreased from 429.7 ± 59.1 µm at baseline to 294 ± 46.5 µm at 6 months (*p* < 0.0001) (Table 2).

Ocular hypertension (30 mmHg) was observed in only one patient during the first week after DEX-I. This condition was well managed with local therapy (dorzolamide/timolol fixed combination two times per day). No other ocular or systemic complications were observed.

A variable increase in CMT after an early reduction was observed in 20 (83%) eyes at a mean time of 2.88 ± 1.65 (3.0 to 6.0) months after DEX-I. But only 3 patients were scheduled for a second DEX-I due to the worsening of CME and BCVA from month 1 to month 6. In one patient, BCVA decreased from 20/100 to 20/200, and CMT increased from 310 μm to 412 μm; in one patient, BCVA decreased from 20/40 to 20/50, and CMT increased from 255 μm to 320 μm; one patient had a visual impairment from 20/200 to 20/317, and a CMT increase from 355 μm to 460 μm.

Univariate regression models revealed a significant relationship between 6-month BCVA and gender (β= −0.27; *p* = 0.03) and macular status when RRD occurred (β = −0.45; *p* = 0.001). No relationship between 6-month CMT and each independent variable was found (Table 3). 

Multiple linear regression model confirmed the relationship between last BCVA and the macular status when RRD occurred (Table 4). 

On the other hand, the same regression model did not reveal any significant relationship between all variables together and 6-month CMT (Table S1).

## 4. Discussion

The results of this study suggested that DEX-I had a good safety profile and significantly improved the anatomical and functional outcomes in eyes that developed an unresponsive CME to medical treatment after a vitrectomy for RRD. To the authors’ knowledge, this is the first study evaluating the safety and effectiveness of 0.7-mg DEX-I performed at the same time as SO removal in vitrectomized eyes for RRD.

Various studies showed that the incidence of post-vitrectomy CME is higher in older patients with pseudophakia or aphakia, macula-off retinal detachment, proliferative vitreoretinopathy (PVR), a high rate of retinotomies, cryotherapy, a high rate of surgeries, and SO endotamponade [3,4,5,9,10,11,12,13]. In our study, the mean age was over 70, 19 (79.1%) patients underwent vitrectomy for macula-off RRD, and different grades of PVR ranging from A to C were observed in all patients. Furthermore, about 70% of patients were pseudophakic, and only SO-filled eyes were selected. 

Chronic CME after RRD repair is thought to be pathophysiologically distinct from other etiologies of CME [7], despite phenotypic similarities [20], similar cytokines involved, and similar tissue responses [6].

If intravitreal corticosteroid therapy revealed its efficacy for managing unresponsive CME [2], also in case of CME occurring after vitrectomy and resistant to topical NSAID therapy [27], the question of whether intravitreal therapy may be less effective in vitrectomized eyes remains. Drug diffusion and clearance from the vitreous cavity are more rapid in vitrectomized eyes, limiting retinal exposure to the drugs and reducing treatment success [28,29]. Nevertheless, the pharmacokinetic profiles [30], the safety profiles, and the clinical outcomes [31] of DEX-I are similar in non-vitrectomized and vitrectomized eyes.

Even though some authors reported spontaneous resolution of CME after SO removal [32], Kiss et al. observed macular changes in 87% of patients with complicated RRD after SO removal, and a percentage of 18% of those patients presented CME that required additional treatment [33].

No data comparing DEX-I with no treatment or different treatments were previously reported, making it challenging to draw conclusions about the first-line therapy for CME following RRD surgery. CADTH has published a recent review on the use of DEX-I for CME after RRD repair. The results have demonstrated statistically significant improvements in BCVA and macular thickness over 6 months. However, the results at 12 months showed a gradual reversion to baseline values of macular thickness and a decrease in visual acuity [24]. Furthermore, Hsu et al. reported that with a single DEX-I, only 25% of eyes showed responsiveness to treatment, 50% of eyes showed no response to the implant, and 25% showed recurrent CME [25]. We also found statistically significant improvement in visual acuity and macular thickness after 6 months from the implant, although a macular thickening following an early reduction in thickness was observed in 83% of patients, and 3 patients were scheduled for a second implant. The recurrence of CME after an early reduction in many patients could suggest that a higher number of patients would have been rescheduled for implants had the follow-up been longer. It is known that SO removal does not eliminate low-molecular weight components that can diffuse from oil to ocular tissue and cause chronic inflammation [34], justifying, in our opinion, the need for prolonged therapeutic coverage, theoretically provided by the implant and its repetition over time.

There have not been studies regarding the factors related to morphologic and functional outcomes after DEX-I to treat recalcitrant CME in SO-filled eyes. Our results revealed that macular-on status had a predictive role in functional recovery after DEX-I for recalcitrant CME. RRD-related macular status has a strong correlation with visual recovery after retinal surgery [35]. In particular, macular OCT biomarkers, including the presence and extension of cystoid edema before surgery and outer retinal layer integrity after surgery, correlate with functional outcomes [35]. Therefore, understandably, the macular sparing that occurs when RRD occurs, preserving the integrity of neuroretinal layers at the central retina, can explain the better functional recovery after RRD repair and also after DEX-I, regardless of the changes in macular thickness. This suggestion may be confirmed by the absence of any correlation between the last CMT and all independent variables.

Although the duration of SO tamponade correlates with CME onset [9,12,13], it seems not to be associated with the outcomes after DEX-I. However, the mean time of macular edema recurrence (about 3 months) was shorter than that commonly reported after the first implant (4–6 months) [36], suggesting that SO removal may not remove all factors of ocular inflammation, thus requiring further implants [33,34]. Furthermore, the duration of macular edema and topical therapy were not associated with outcomes after the implant. The association between efficacy outcomes and the severity of macular edema or persistence is still controversial considering different conditions such as diabetic retinopathy [37] or vein occlusion [38]. Furthermore, the topical drugs (diclofenac sodium and betamethasone) used are probably not the most effective overall, but they are the most effective at our disposal.

In all previous studies, no safety issues were observed with DEX-I [36,37,38,39,40,41]; in some cases, an increase in IOP occurred, but it was rarely above the normal range and was easily managed with topical therapy [36,37,38,39,40,41], as we observed. Furthermore, we chose a cut-off of ocular hypertension similar to that reported in the Phase III registration study of DEX-I [42], but higher than that considered in some studies on previously vitrectomized eyes, considering that a higher rate of ocular hypertension was found in vitrectomized eyes compared with nonvitrectomized ones [43], and the implant could get worse in this condition.

With regard to the safety of surgical technique, the implant injection was performed when the eye was filled with BSS to avoid possible retinal trauma due to the implant’s kinetic energy, as previously suggested [44].

The main limitations of our study are its retrospective, single-center design and the relatively small number of patients; the short follow-up does not allow observation of possible recurrences of CME; the absence of analysis of OCT biomarkers at baseline, which is potentially able to influence functional and morphological recovery after DEX-I. Moreover, another limitation of the study is the absence of adjustments for multiplicity, and as such, all analyses should be regarded as exploratory.

In conclusion, DEX-I during SO removal is an effective and safe approach to successfully treat refractory to medical therapy macular edema after RRD surgery. Further studies are needed on a larger sample and a longer follow-up to evaluate the rate of CME recurrence, the rate of retreatment with DEX-I, and OCT findings of neuroretinal layers with their relationship with functional recovery.

## Figures and Tables

**Table 1:** Anamnestic and clinical characteristics (*n* = 24).

Parameters *	Mean ± SD or %
Age (yrs)	71.71 ± 7.91
Gender (M) (%)	16 (66.67)
Glaucoma (Yes) (%)	5 (20.83)
Lens Status (%)	
Phakic	7 (29.17)
Pseudophakic	17 (70.83)
RRD Macula (%)	
Off	19 (79.17)
On	5 (20.83)
PVR (Grade A-C)	24 (100%)
Time between PPV and CME onset, days	27.42 ± 7.78
Duration of CME Topical Therapy, days	95.58 ± 5.76
Time between PPV and DEX-I, days	99.13 ± 5.41
Complication	
None	23 (95.83)
IOP ≥ 25 mmg	1 (4.17)

* As Mean and Standard Deviation (SD) for continuous variables, and frequency and percentage (%) for categorical; RRD, Rhegmatogenous retinal detachment; PVR: proliferative vitreoretinopathy; PPV: Pars plana vitrectomy; CME; Cystoid macular edema; DEX-I, intravitreal dexamethasone implant; IOP, intraocular pressure.

**Table 2:** Changes of outcomes over follow-up.

	Baseline	1 Month	3 Months	6 Months
BCVA (LogMAR)	0.99 ± 0.3	0.62 ± 0.3	0.62 ± 0.3	0.60 ± 0.3
*p* ^§^		<0.0001	<0.0001	<0.0001
CMT (µm)	429.6 ± 59.1	271.8 ± 28.4	287.9 ± 32.4	294.0 ± 46.4
*p* ^§^		<0.0001	<0.0001	<0.0001

BCVA: Best-corrected Visual Acuity, CMT: Central Macular Thickness; ^§^ Wilcoxon matched-pairs signed-rank test.

**Table 3:** Univariate regression models of BCVA and OCT at 6 months after DEX-I, on demographic and clinical parameters.

Parameters	β	se (β)	*p*	C.I. (95%)
6m Post DEX-I BCVA				
Gender (w)	−0.27	0.12	0.03	−0.52 to −0.02
Age (yrs)	−0.002	0.01	0.78	−0.02 to 0.01
Glaucoma (Yes)	0.03	0.15	0.85	−0.29 to 0.35
Pseudophakia	−0.01	0.14	0.97	−0.29 to 0.28
Days between PPV and CME onset	−0.003	0.01	0.69	−0.02 to 0.01
Days of topic therapy	−0.01	0.01	0.27	−0.03 to 0.01
Days between PPV and DEX-I	−0.001	0.01	0.72	−0.01 to 0.01
RRD Macula (On)	−0.45	0.12	0.001	−0.70 to −0.19
6m Post DEX-I CMT				
Gender (w)	−16.87	20.26	0.41	−58.90 to 25.15
Age (yrs)	0.74	1.24	0.56	−1.84 to 3.32
Glaucoma (Yes)	31.83	22.90	0.18	−15.67 to 79.33
Pseudophakia	−4.03	21.33	0.85	−48.26 to 40.19
Days between PPV and CME onset	−1.75	1.21	0.16	−4.28 to 0.76
Days of topic therapy	−0.70	1.75	0.68	−4.26 to 2.85
Days between PPV and DEX-I	−0.67	1.82	0.71	−4.46 to 3.11
RRD Macula (On)	−21.47	23.44	0.37	−70.10 to 27.15

Abbreviations: DEX-I, intravitreal dexamethasone implant; BCVA, Best-corrected visual acuity; PPV, Pars Plana Vitrectomy; CME: Cystoid macular edema; CMT, Central macular thickness; RRD, Regmatogenous Retinal detachment; β, Coefficient; se (β), Standard Error of β; C.I. (95%), Confidential Interval at 95%.

**Table 4:** Multiple linear regression model of BCVA at 6 months after DEX-I and all variables together in the model.

Parameters	β	se (β)	*p*	C.I. (95%)
6m Post DEX-I BCVA				
Gender	−0.18	0.14	0.21	−0.48 to 0.11
Age (yrs)	0.0001	0.01	0.98	−0.02 to 0.02
Glaucoma	0.03	0.14	0.81	−0.26 to 0.33
Pseudophakia	−0.03	0.20	0.87	−0.47 to 0.41
Days between PPV and CME onset	−0.005	0.008	0.46	−0.02 to 0.01
Days of topic therapy	0.01	0.04	0.72	−0.08 to 0.11
Days between PPV and DEX-I	−0.02	0.04	0.64	−0.11 to 0.07
RRD Macular Status	−0.38	0.14	0.02	−0.70 to −0.07

Abbreviations: DEX-I, intravitreal dexamethasone implant; BCVA, Best-corrected visual acuity; PPV, Pars Plana Vitrectomy; CME: Cystoid macular edema; CMT, Central macular thickness; RRD, Regmatogenous Retinal detachment; β, Coefficient; se (β), Standard Error of β; C.I. (95%), Confidential Interval at 95%.

## Data Availability

The data that support the findings of the present study are available from the corresponding author (A.N.) upon reasonable request.

## Supplementary Materials

The following supporting information can be downloaded at: https://www.mdpi.com/article/10.3390/jcm12041697/s1. Table S1: Multiple linear regression model of CMT at 6 months after DEX-I and all variables together in the model.
