# Supplementary material for: Intravitreal Dexamethasone Implant at the Time of Silicon Oil Removal to Treat Persistent Macular Edema after Rhegmatogenous Retinal Detachment Repair
Source: J Clin Med. 2023 Feb 20;12(4):1697. doi: 10.3390/jcm12041697 (PMC9962319; doi:10.3390/jcm12041697)
Supplement: Supplementary file 1 [file jcm-12-01697-s001.zip › jcm-2171672-supplementary.pdf]

Table S1. Multiple linear regression model of CMT at 6 months after DEX-I and all variables together in the model.

**6m Post DEX-I CMT**				
Gender (w)	-16.87	20.26	0.41	-58.90 to 25.15
Age (yrs)	0.74	1.24	0.56	-1.84 to 3.32
Glaucoma (Yes)	31.83	22.90	0.18	-15.67 to 79.33
Pseudophakic	-4.03	21.33	0.85	-48.26 to 40.19
Days between PPV and CME onset	-1.75	1.21	0.16	-4.28 to 0.76
Days of topic therapy	-0.70	1.75	0.68	-4.26 to 2.85
Days between PPV and DEX-I	-0.67	1.82	0.71	-4.46 to 3.11
RRD Macula (On)	-21.47	23.44	0.37	-70.10 to 27.15
